# Post‐translational modifications in capsid proteins of recombinant adeno‐associated virus (AAV) 1‐rh10 serotypes

**DOI:** 10.1111/febs.15013

**Published:** 2019-08-01

**Authors:** Bertin Mary, Shubham Maurya, Sathyathithan Arumugam, Vikas Kumar, Giridhara R. Jayandharan

**Affiliations:** ^1^ Department of Biological Sciences and Bioengineering Indian Institute of Technology Kanpur India; ^2^ SASTRA University Thanjavur India; ^3^ Mass Spectrometry and Proteomics Core Facility University of Nebraska Medical Center Omaha NE USA; ^4^ Department of Haematology and Centre for Stem Cell Research Vellore India

**Keywords:** AAV, capsid, LCMS, MALDI‐TOF analysis, post‐translational modification

## Abstract

Post‐translational modifications in viral capsids are known to fine‐tune and regulate several aspects of the infective life cycle of several viruses in the host. Recombinant viruses that are generated in a specific producer cell line are likely to inherit unique post‐translational modifications during intra‐cellular maturation of its capsid proteins. Data on such post‐translational modifications in the capsid of recombinant adeno‐associated virus serotypes (AAV1‐rh10) is limited. We have employed liquid chromatography and mass spectrometry analysis to characterize post‐translational modifications in AAV1‐rh10 capsid protein. Our analysis revealed a total of 52 post‐translational modifications in AAV2‐AAVrh10 capsids, including ubiquitination (17%), glycosylation (36%), phosphorylation (21%), SUMOylation (13%) and acetylation (11%). While AAV1 had no detectable post‐translational modification, at least four AAV serotypes had >7 post‐translational modifications in their capsid protein. About 82% of these post‐translational modifications are novel. A limited validation of AAV2 capsids by MALDI‐TOF and western blot analysis demonstrated minimal glycosylation and ubiquitination of AAV2 capsids. To further validate this, we disrupted a glycosylation site identified in AAV2 capsid (AAV2‐N253Q), which severely compromised its packaging efficiency (~ 100‐fold vs. AAV2 wild‐type vectors). In order to confirm other post‐translational modifications detected such as SUMOylation, mutagenesis of a SUMOylation site(K258Q) in AAV2 was performed. This mutant vector demonstrated reduced levels of SUMO‐1/2/3 proteins and negligible transduction, 2 weeks after ocular gene transfer. Our study underscores the heterogeneity of post‐translational modifications in AAV vectors. The data presented here, should facilitate further studies to understand the biological relevance of post‐translational modifications in AAV life cycle and the development of novel bioengineered AAV vectors for gene therapy applications.

**Enzymes:**

Trypsin, EC 3.4.21.4

## Graphical abstract

The capsids of adeno‐associated virus (AAV) vectors are composed of three proteins (VP1,VP2, and VP3). During its assembly and packaging in human cells, AAV capsids may acquire diverse post‐translational modifications. Our study has analysed the spectrum of post‐translational modifications in AAV1‐rh10 serotype capsids and highlights the type and heterogeneity of such modifications between serotypes. This information is also useful to understand the biology of these post‐translational modifications in the viral life cycle and has further defined targets for AAV capsid bioengineering.

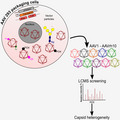

**Abbreviations**
AAV: adeno‐associated virus
PTM: post‐translational modification
AAP: assembly activating protein
ORF: open reading frame
PLA2: phospholipase A2
NLS: nuclear localization signal
EGFP: enhanced green fluorescence protein
PBS: phosphate buffered saline
QPCR: quantitative polymerase chain reaction
DTT: dithiothreitol
MALDI‐TOF: matrix‐assisted laser desorption/ionization time of flight
LC/MS: liquid chromatography–mass spectrometry
PCM: polydimethylcyclosiloxane
HIV: human immunodeficiency virus
SUMO: small ubiquitin like modifier

## Introduction

Adeno‐associated virus (AAV), are single‐stranded DNA viruses of the *Parvoviridae* family and have become an excellent choice for gene therapy 1, 2, 3, 4 due to their non‐pathogenic nature and a relatively stable therapeutic gene expression 5, 6. The translational potential of AAV has further benefited from the repertoire of multiple serotypes available [AAV1‐rh10] 7, 8. Each of these AAV serotypes has a unique capsid sequence that defines their interactions with host cells 9, 10, 11. The capsid of AAV is icosahedral in shape and is composed of 60 subunits of three viral capsid proteins VP1, VP2, and VP3 in the ratio of 1:1:10, while the assembly activating protein (AAP) aids in viral capsid assembly 12, 13, 14. These viral proteins share a common C‐terminal region, but are generated by alternative splicing of the right open reading frame (ORF) of AAV genome. Differences in sequence homology of the structural proteins between the AAV serotypes, confers them unique binding affinity toward various host cell surface receptors 15, 16. Several other regions of the viral capsid are to known to have evolved to manipulate host cellular processes in order to establish a successful infection. The N‐terminus of VP1 contains a conserved phospholipase‐A2 (PLA2) domain, which plays an important role in the endosomal escape of virus 17. Furthermore, several nuclear localization signals (NLS) have been reported within the N terminus of VP1 and VP2 of the capsid that are recognized by the cellular transport machinery to facilitate nuclear transport 18. In addition, sequence differences within the VP3 region, are known to contribute to the diverse tissue tropism of AAV vectors. For example, in AAV2 the heparin binding site formed by positively charged R585, R588, and R487 residues 19, 20, allows the virions to bind the heparan sulfate proteoglycan cellular surface receptor and initiate the transduction process 21. Mutational disruption of these residues correlates with a decline in heparin/cell binding property and a loss of transduction 22, 23.

While the role of functional domains of the VP1‐3 proteins and receptor foot‐prints are known for certain AAV serotypes (AAV1‐6, 9) 9, 11, 23, 24, 25, the impact of the post‐translational modifications (PTM) of the capsid proteins that may fine tune several aspects of viral infectivity and dictate systemic host response, is poorly understood. PTMs are functional modifications that occur on specific amino acid residues after protein translation. This may include biochemical or covalent attachment of a phosphate group (Phosphorylation), ubiquitin protein (Ubiquitination), sugar moities (Glycosylation), SUMO‐1/2/3 protein (SUMOylation), acetyl group (Acetylation), etc. 26. PTM during viral protein synthesis in a host cell is an important source of generating structural and functional diversity/complexity that enables the virus to adapt and co‐evolve to the various host cellular barriers as demonstrated earlier for Adenovirus, Vaccinina virus and Polyoma virus 27, 28, 29. In case of recombinant vectors such as AAV, they are packaged *in vitro* under a multitude of conditions including the type and concentration of plasmid DNA used 30 **,** the type of producer cell lines employed (293 cells or insect cell, sf9 based or HeLa cells) 31, 32, 33. Furthermore, in the triple transfection protocol for vector production that is commonly used for packaging of AAV 34, the synthesis and expression of non‐structural and structural components of AAV involves the host protein synthesis and its maturation machinery. Therefore, it stands to reason that during packaging of AAV, the capsid proteins are likely to inherit PTMs whose level and type may vary based on the serotype that is packaged. Such modifications in recombinant AAV1‐rh10 if any, is likely to be an inherent determinant of its infectious cycle in the host.

**Figure 1:**
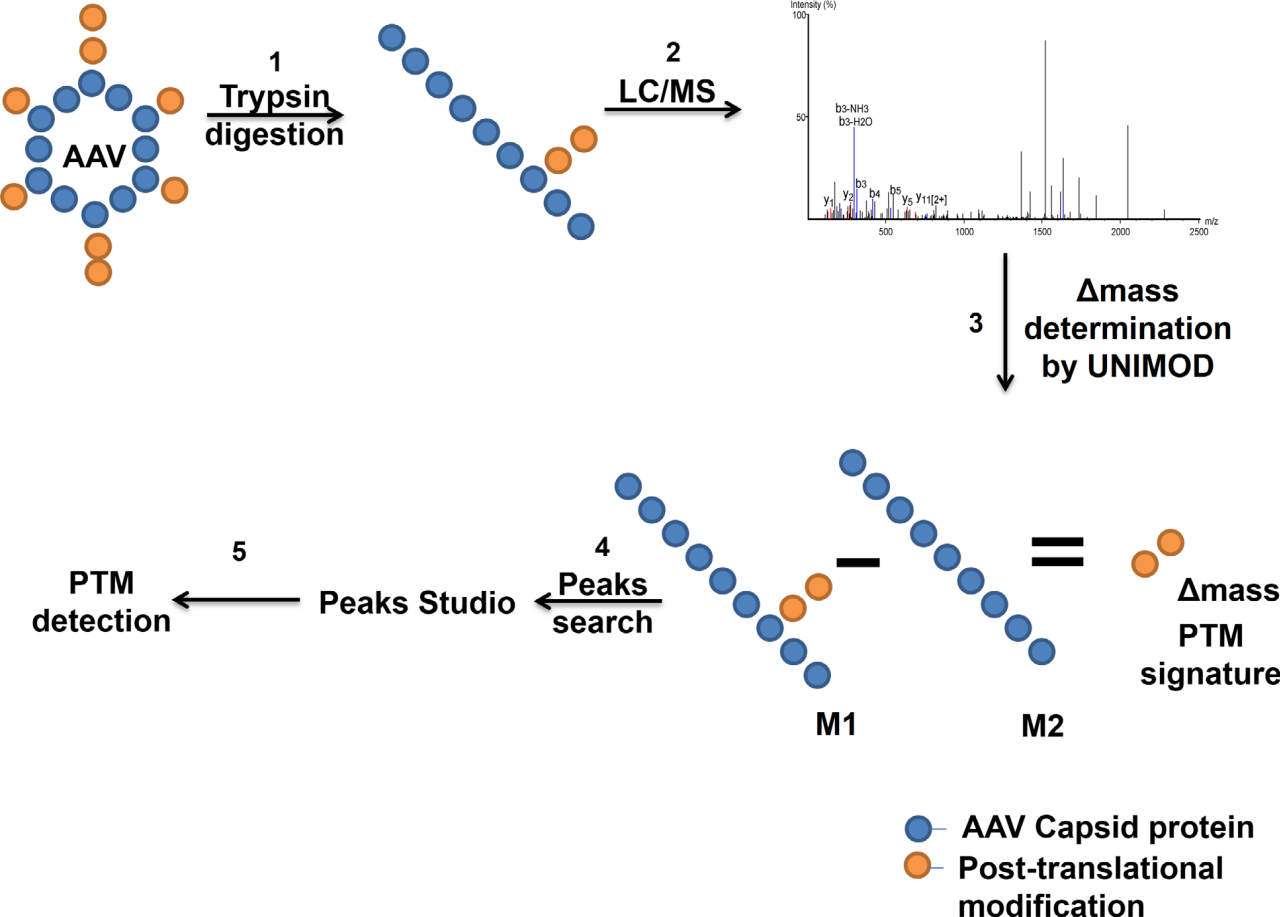
Experimental set‐up for detection of post‐translational modification on AAV capsids. (1) AAV capsids were digested in solution with trypsin (2) followed by analysis with nano ultra‐performance liquid chromatography (UPLC) coupled with Triple time of flight‐mass spectrometry (Triple TOF‐MS) analysis, (3) Δmass values were obtained from UNIMOD. (4) Data obtained between putative modified (M1) and reference peptide sequences (M2) were analyzed by PEAKS Studio 8.0. (5) Y‐ion and B‐ion spectra validation was performed to consolidate the post‐translational modifications (PTM) identified.

Several studies have demonstrated the rate‐limiting effects of these capsid specific PTMs. These sites have been largely identified by *in silico* predictions. For e.g., mutations in AAV capsid specifically targeting phosphorylation sites including tyrosine 35, serine, and threonine residues 36 or ubiquitination of lysine residues have improved the transduction of mutated AAV *in vitro* and *in vivo* 36, 37, 38, 39. However, experimental evidence on the presence of PTMs in *Rep* or *Cap* proteins, particularly post‐packaging of recombinant AAVs is limited. Weger *et al*. 40 demonstrated that AAV *rep* proteins Rep68 and Rep78 are modified by SUMOylation process, thus contributing to its stability. In a prototype strain of Parvovirus minute virus of mice, the phosphorylation of serine and threonine residues of capsid components has been demonstrated after its assembly by ^32^P‐ pulse‐chase studies 41. In case of AAV generated with live Adenovirus [Ad2] 33, Murray *et al*. 42 performed Fourier transform ion cyclotron mass spectrometry analysis of AAV packaged in HeLa cells and demonstrated a lower to negligible level of glycosylation in AAV2. Similarly, mass spectrometry based analysis of the mass of AAV1 and AAV8 demonstrated a considerable difference in the stoichiometry of VP1, VP2, and VP3 proteins and the heterogeneous nature of the capsids between these serotypes 43, 44. In case of recombinant AAV2 generated by the triple transfection protocol in HEK293 cells, a recent study demonstrated acetylation of capsid proteins at the N‐terminal region corresponding to a +42 Da mass change in liquid chromatography‐mass spectrometry (LC/MS) analysis. This acetylation PTM of the capsid was further conserved in AAV1, AAV2, AAV5, AAV7, AAV9, and rh10 vectors 16. Furthermore, the capsid protein diversity due to another PTM, the deamidation of Asparagine residue was recently reported in AAV1, AAV3B, AAV4, AAV5, AAV7, AAV8, AAV9, and Rh32.33 serotypes 45. Interestingly, in AAV8 serotype, a single aminoacid has been associated with two PTMs, the N‐glycosylation and deamidation of N499 residue 45, 46. This highlights the heterogeneity of PTM modifications in capsid proteins, which may be due to variability between packaging lots as described by Giles *et al*. 45. Given the paucity of data on other PTM modifications in AAV capsids 16, 42, the present study was designed to comprehensively study the global PTM profile of the common AAV serotypes 1‐rh10, using advanced mass spectrometry‐based approach (Fig. 1) and to further perform limited validation of select PTMs that were identified in our screening.

## Results

### Glycan profiling of AAV2 capsid by MALDI‐TOF/TOF analysis

**Figure 2:**
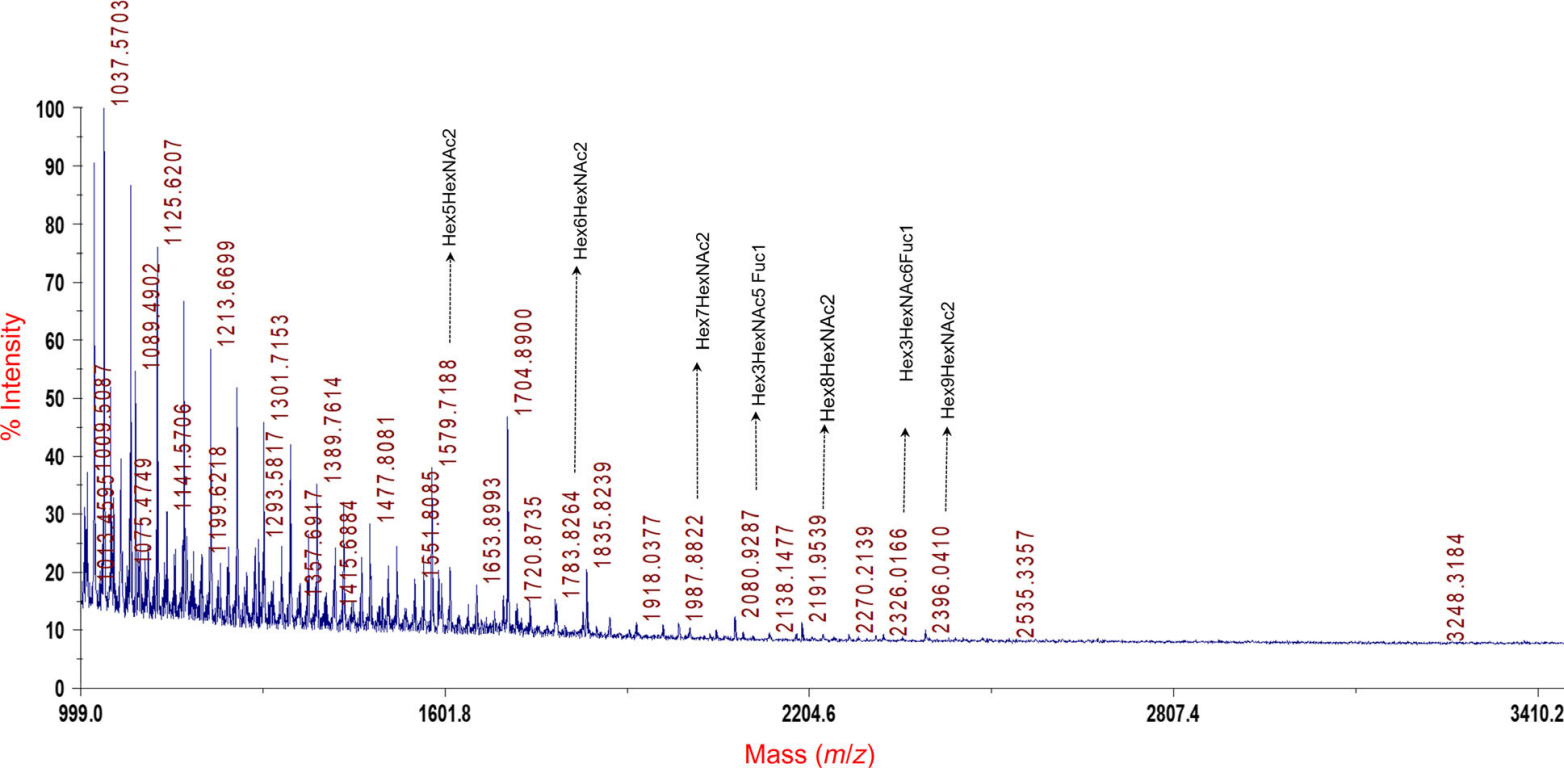
Representative spectra of matrix‐assisted laser desorption/ionization time of flight (MALDI‐TOF) analysis done for glycan characterization on AAV2 capsid protein (*n* = 2 replicates). A few of the glycan combinations are highlighted in the figure to their approximate mass (m/z) values, while their complete list is provided in Table 1.

To identify possible glycans in the AAV capsids, we first performed a MALDI‐TOF/TOF analysis on the prototype vector, AAV2. Our initial screening was aimed to detect N‐linked glycans on AAV2 capsid after its digestion with PNGase F. The MALDI spectra obtained showed several ions within a mass range of m/z 1000–3000. Our data analysis of the spectra identified 10 possible N‐ glycan combinations on AAV2 capsid with variable m/z values (Fig. 2). The elemental combination of these glyco‐forms along with their m/z values and monoisotopic mass is presented in Table 1.

**Table 1:** N‐linked glycans identified on AAV2 capsid by matrix‐assisted laser desorption/ionization time of flight (MALDI‐TOF) analysis.

Possible combination of N‐glycan	m/z	Elemental Composition	Mono mass	Average mass
Hex7HexAc2	1987.8	C_58_O_45_N_2_H_96_	1540.5284	1541.36
Hex3HexNAc4Fuc1	2039.8	C_56_O_39_N_4_H_92_	1444.5339	1445.32
Hex3HexNAc5Fuc1	2080.9	C_64_O_44_N_5_H_105_	1647.6133	1648.51
Hex8HexNAc2	2192.0	C_64_O_50_N_2_H_106_	1702.5812	1703.5
Hex3HexNAc6Fuc1	2326.0	C_72_O_49_N_6_H_118_	1850.6927	1851.70
Hex9HexNAc2	2396.0	C_70_O_55_N_2_H_116_	1864.6340	1865.64
Hex4HexNAc2	1375.6	C_40_O_30_N_2_H_66_	1054.37	1054.94
Hex5HexNAc2	1579.7	C_46_O_35_N_2_H_76_	1216.4228	1217.08
Hex3HexNAc3Fuc1	1590.7	C_48_O_34_N_3_H_79_	1241.4545	1242.13
Hex6HexNAc2	1783.7	C_52_O_40_N_2_H_86_	1378.4756	1379.22

Subsequently, we performed a MALDI‐TOF/TOF screening for O‐linked glycan modifications after per methylation of capsid protein with methyl iodide. Our analysis did not detect any O‐glycan on the AAV2 capsid, possibly due to technical limitations of the MALDI analysis. Nonetheless these data suggest that AAV2 capsid is N‐glycosylated.

### Identification of post‐translational modification in AAV capsids by LC/MS

To determine the presence of PTMs on AAV capsids, we performed nanoUPLC analysis coupled with Triple‐TOF MS analysis of AAV1 to AAVrh10 serotypes after its enzymatic digestion with trypsin. This PTM screening utilized two biological replicate samples of AAV1‐rh10 vectors, each of which were analysed twice by LC/MS. A putative PTM signature was defined based on the differential ∆mass values of modified and unmodified peptides generated by trypsin digestion in any of these samples screened. The ∆mass values for PTMs were obtained from UNIMOD (http://www.unimod.org/) if required 47, 48 (Table 2). A manual validation was performed in addition to software assignment to ensure correct peptide identification. Our analysis showed the presence of a total of 52 PTMs including acetylation, phosphorylation, ubiquitination, SUMOylation of different glycans in AAV2, AAV3, AAV4, AAV5, AAV6, AAV7, AAV8, AAV9, and AAVrh10 capsids (Table 3). Among the different serotypes studied, AAV1 had no detectable PTM, while AAV4, AAV6, and AAV8 were the least modified vectors (1–3 PTMs), whereas AAV2, AAV5, AAV9, and AAVrh10 showed multiple PTM modifications (≥7 PTMs) (Table 4). Among the type of PTMs detected in AAV capsids, glycosylation (36%, *n* = 19/52) and phosphorylation (21%, *n* = 11/52) were common than other modifications (Table 4).

**Table 2:** Δmass values obtained from UNIMOD database for post‐translationally modified proteins after trypsin digestion.

Post‐translational modification	Δmass
Acetylation (Protein N‐term)	+42.01
Phosphorylation	+79.97
Ubiquitination	+114.04
SUMOylation by SUMO‐1	+600.25
SUMOylation by SUMO‐2/3	+599.27

**Table 3:** Post‐translational modifications present on AAV2‐AAVrh10 serotypes after liquid chromatography and mass‐spectrometry (LC/MS) and PEAKS analysis. ***Ph***‐Phosphorylation, ***Ub***‐Ubiquitination, ***Hx***‐HexNAcylation, ***Ac***‐Acetylation, ***Ng***‐N‐linked glycan core, ***Su***‐ SUMO‐1 or SUMO‐2/3.

Serotype	Site	Modification	Peptide sequence
AAV2	S149^a^	Phosphorylation	RPVEH**S**_***Ph***_PVEPDSSSGTGK
	N253	Hex(4) HexNAc(2)	TWALPTY**N**_***Ng***_NHLYKQIS
	K258	SUMOylation by SUMO‐2/3	TWALPTYNNHLY**K**_***Su***_QIS
	N518	HexNacylation	LNGRDSLV**N**_***Hx***_PGPAMASHKDDEEK
	S537	dHex Hex(3) HexNAc(6)	EKFFPQ**S**_***Ng***_GVLIFGK
	K544^a^	Ubiquitination	SGVLIFG**K**_***Ub***_
	N551	HexNAcylation	KT**N**_***Hx***_VDIEKVMITDEEEIR
AAV3	A2^a^	Acetylation	**A**_***Ac***_ADGYLPDWLEDNLSEGIR
	Y6^a^	Phosphorylation	AADG**Y**_***Ph***_LPDWLEDNLSEGIR
	S149^a^	HexNAcylation	KKGAVDQ**S**_***Hx***_PQEPDSSSGVGK
AAV4	K479	Ubiquitination	KNWLPGPSI**K**_***Ub***_QQGFSK
AAV5	K122^a^	Ubiquitination	KAVFQAK**K**_***Ub***_R
	N292^a^	N‐linked Glycan core	RLI**N**_***Ng***_NYWGFRPRS
	N308^a^	HexNAcylation	KIF**N**_***Hx***_IQVKE
	K312	SUMOylation by SUMO‐2/3	VKIFNIQV**K**_***Su***_
	T376^a^	HexNAcylation	RDN**T**_***Hx***_ENPTERS
	N428	dHex Hex(3) HexNAc(5)	LA**N**_***Ng***_PLVDQYLYR
	K451^a^	Ubiquitination	RFVSTNNTGGVQFN**K**_***Ub***_N
	K639^a^	Ubiquitination	KHPPPMMLI**K**_***Ub***_N
	S680^a^	Phosphorylation	KEN**S**_***Ph***_KRW
AAV6	A2^a^	Acetylation	M**A**_***Ac***_ADGYLPDWLEDNLSEGIRE
AAV7	A2^a^	Acetylation	M**A**_***Ac***_ADGYLPDWLEDNLSEGIRE
	K61^a^	SUMOylation by SUMO‐2/3	KYLGPFNGLD**K**_***Su***_GEPVNAADAAALEHDKA
	S157	HexNAcylation	SPDS**S**_***Hx***_TGIGKKGQQPARK
	T252	Phosphorylation	TWALP**T**_***Ph***_YNNHLYK
	N254	HexNAcylation	TWALPTY**N**_***Hx***_NHLYK
	N460	HexNAcylation	TQSNPGGTAG**N**_***Hx***_R
AAV8	A2^a^	Acetylation	M**A**_***Ac***_ADGYLPDWLEDNLSEGIRE
	Y6^a^	Phosphorylation	MAADG**Y**_***Ph***_LPDWLEDNLSEGIRE
	N521	N‐linked Glycan core	NSLA**N**_***Hx***_PGIAMATHK
AAV9	A2^a^	Acetylation	M**A**_***Ac***_ADGYLPDWLEDNLSEGIRE
	Y52^a^	Phosphorylation	K**Y**_***Ph***_LGPGNGLDKG
	N57^a^	HexNAcylation	KYLGPG**N**_***Hx***_GLDKG
	K84^a^	SUMOylation by SUMO‐1	AYDQQL**K**_***Su***_A
	K105	Ubiquitination	L**K**_***Ub***_EDTSFGGNLGR
	K316	SUMOylation by SUMO‐2/3	RLNF**K**_***Su***_
	T450	Phosphorylation	**T**_***Ph***_INGSGQNQQTLK
	S454^a^	Phosphorylation	TING**S**_***Ph***_GQNQQTLK
	K557	SUMOylation by SUMO‐1	DNVDAD**K**_***Su***_
	K650^a^	Ubiquitination	HPPPQILI**K**_***Ub***_N
	T702^a^	dHex Hex(3) HexNAc(3)	RWNPEIQY**T**_***Ng***_SNYYK
AAVrh10	A2^a^	Acetylation	**A**_***Ac***_ADGYLPDWLEDNLSEGIR
	Y90	Phosphorylation	AGDNP**Y**_***Ph***_LR
	S149^a^	Phosphorylation	TAPGKKRPVEP**S**_***Ph***_PQR
	S157	HexNAcylation	SPDS**S**_***Hx***_TGIGK
	T252	Phosphorylation	TWALP**T**_***Ph***_YNNHLYK
	N306	N‐linked glycan core	LINN**N**_***Ng***_WGFRPK
	T332^a^	HexNAcylation	EVTQNEG**T**_***Hx***_KT
	K333^a^	Ubiquitination	EVTQNEGT**K**_***Ub***_T
	S449	HexNAcylation	LMNPLIDQYLYYL**S**_***Hx***_R
	K652	Ubiquitination	HPPPQILI**K**_***Ub***_
	K709	SUMOylation by SUMO‐2/3	RWNPEIQYTSNYY**K**_***Su***_

^a^ Denotes modification site identified by spectra in multiple runs (>1 run).

**Table 4:** Distribution of post‐translational modifications detected in AAV1‐rh10 serotypes.

Serotype	Ubiquitination	Phosphorylation	SUMOylation	Glycosylation	Acetylation	Total PTMs
AAV1	–	–	–	–	–	0
AAV2	1	1	1	4	–	7
AAV3	–	1	–	1	1	3
AAV4	1	–	–	–	–	1
AAV5	3	1	1	4	–	9
AAV6	–	–	–	–	1	1
AAV7	–	1	1	3	1	6
AAV8	–	1	–	1	1	3
AAV9	2	3	3	2	1	11
AAVrh10	2	3	1	4	1	11
Total no. of sites	9	11	7	19	6	52
Abundance of PTMs in AAV	17.30%	21.15%	13.46%	36.53%	11.53%	

**Figure 3:**
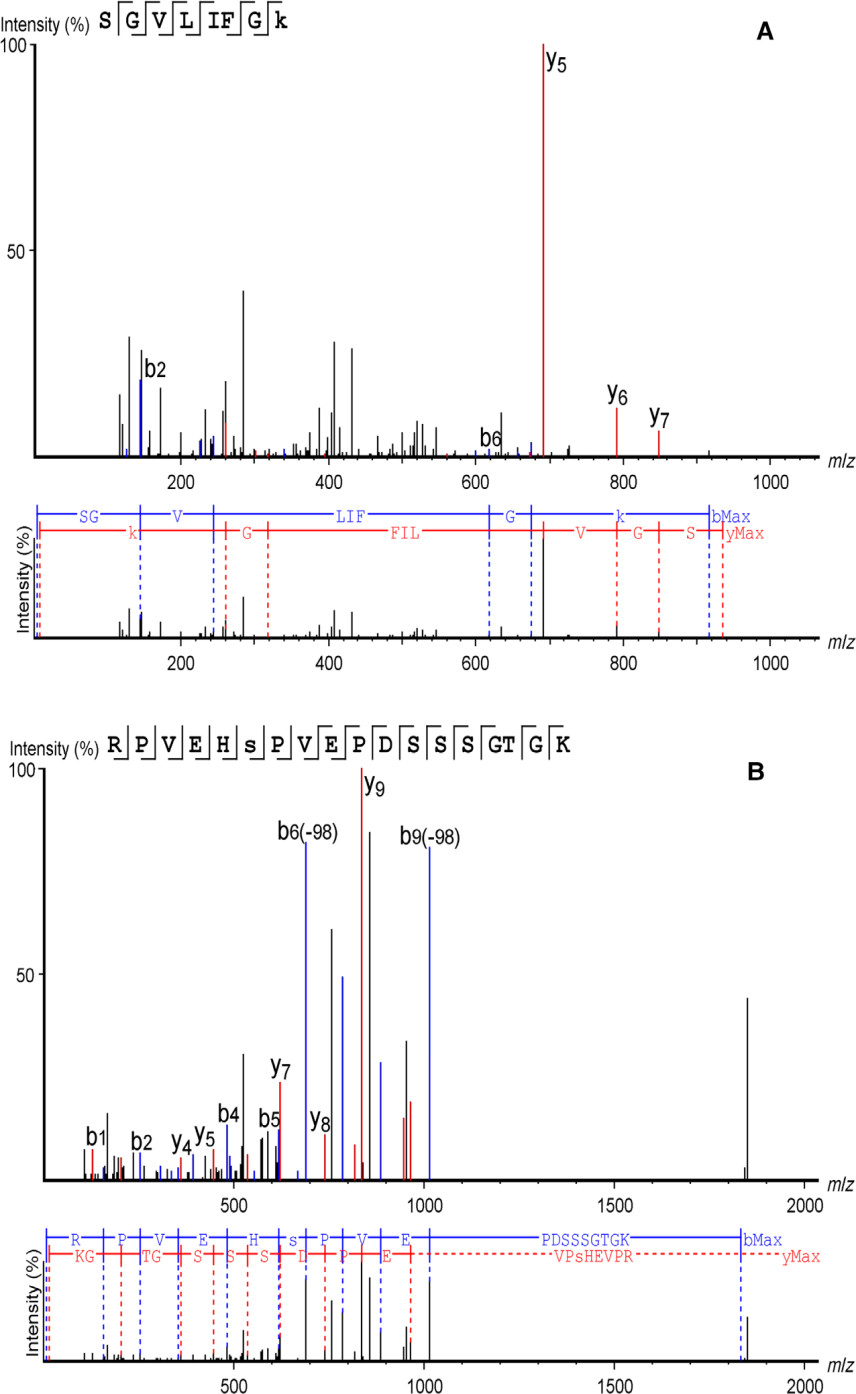
Representative spectra identified in AAV2 capsid for ubiquitination modification at residue K544 (*n* = 2 replicates) (A) and phosphorylation modification at S149 residue (*n* = 4 replicates) (B). Mass spectrometry data of trypsin digested peptides from AAV2 was acquired on QExactive and analyzed using PEAKS studio 8.0. Peptide identification and modification assignment was performed from tandem mass‐spectrometry of the selected precursors.

AAV2 capsid showed the presence of ubiquitination at codon K544, phosphorylation at S149, SUMOylation by SUMO‐2/3 at K258. Interestingly, residues N253, N518, S537, and N551 with detectable PTMs in AAV2 were glycosylated. A previous study by Li *et al*., 2015 had predicted K544 as one of the target site for ubiquitination by *in silico* prediction based on solvent accessibility 49 (Table 5). Mutagenesis of the target site from Lysine to Glutamate at codon 544 led to a decreased proteosome‐mediated degradation of the capsid and resulted in higher transduction efficiency 36. Furthermore, PEAKS analysis also detected the presence of glycosylation of AAV2 capsids at residues N253, N518, S537, and N551. N518 and N551 sites are found to be HexNAcylated with a mass change +203.08 Da, whereas N253 is modified by complex glycan structure Hex(4)HexNAc(2) and S537 by dHexHex(3)HexNAc(6). Representative spectra for ubiquitination at K544 and phosphorylation at residue S149 are denoted in Fig. 3.

**Table 5:** Post‐translational modification sites on AAV serotypes that have been previously predicted/detected and further validated in our liquid chromatography‐mass spectrometry (LC/MS) protocol.

Serotype	Modification/Site	Method	References
AAV2	K258E	Lysine elimination	49
	S537A	Prediction Method	36
	K544E	Lysine elimination	49
AAV4	K479N	Random library Generation	75
AAV7	A2	LC/MS	16
AAV9	A2	LC/MS	
AAVrh10	A2	LC/MS	
	T252A	Prediction based	50
	K333R	Prediction based	

In case of AAV3, an N‐acetylation modification was seen at the A2 position with a +42 Da mass change. Also, phosphorylation and HexNAcylation was observed in residues Y6 and S149 respectively. In AAV4, a ubiquitination site at K479 was identified and this modification was unique and absent in all other serotypes analysed. Interestingly, several PTMs (*n* = 9) were observed in the most divergent serotype, AAV5. A majority (*n* = 7) of these were glycosylation or ubiquitination modifications‐ including HexNAcylation at residues N308, T376, N428, presence of glycan core at N292 residue and ubiquitination detected at K122, K451, and K639. On the other hand, AAV6 had a single acetylation at residue A2. Surprisingly, a higher proportion (50%) of glycosylation PTMs were identified in AAV7 (S157, N254, N460). In AAV8, a serotype that has tremendous translational potential had only three PTMs identified, a phosphorylation at Y6 residue, acetylation of A2 residue, and an N‐linked glycan core at N521 residue. Both AAV9 and AAVrh10 had a similar distribution of the common PTMs identified, with a relatively higher proportion of sumoylated residues detected in AAV9 (K84, K316, K557) in comparison to all other serotypes. The complete profile of other PTMs in all the AAV serotypes is detailed in Table 3. The number and type of PTMs detected here is quite varied and a majority have not been described previously (Table 6). These data should serve as a wealth of resource for further studies to improve the phenotype of these alternate serotypes.

**Table 6:** Novel post‐translational modification sites detected in AAV serotypes by liquid chromatography‐mass spectrometry (LC/MS) analysis.

Serotype	Site	Modification
AAV2	S149	Phosphorylation
	N253	Hex(4) HexNAc(2)
	N518	HexNAcylation
	N551	HexNAcylation
AAV3	A2	Acetylation
	Y6	Phosphorylation
	S149	HexNAcylation
AAV5	K122	Ubiquitination
	N292	N‐lined Glycan core
	N308	HexNAcylation
	K312	SUMOylation by SUMO‐2/3
	T376	HexNAcylation
	N428	dHex Hex(3) HexNAc(5)
	K451	Ubiquitination
	K639	Ubiquitination
	S680	Phosphorylation
AAV6	A2	Acetylation
AAV7	K61	SUMOylation by SUMO‐2/3
	S157	HexNAcylation
	T252	Phosphorylation
	N254	HexNAcylation
	N460	HexNAcylation
AAV8	A2	Acetylation
	Y6	Phosphorylation
	N521	N‐linked Glycan core
AAV9	Y52	Phosphorylation
	N57	HexNAcylation
	K84	SUMOylation by SUMO‐1
	K105	Ubiquitination
	K316	SUMOylation by SUMO‐2/3
	T450	Phosphorylation
	S454	Phosphorylation
	K557	SUMOylation by SUMO‐1
	K650	Ubiquitination
	T702	dHex Hex(3) HexNAc(3)
AAVrh10	Y90	Phosphorylation
	S149	Phosphorylation
	S157	HexNAcylation
	N306	N‐linked Glycan core
	T332	HexNAcylation
	S449	HexNAcylation
	K652	Ubiquitination
	K709	SUMOylation by SUMO‐2/3

**Figure 4:**
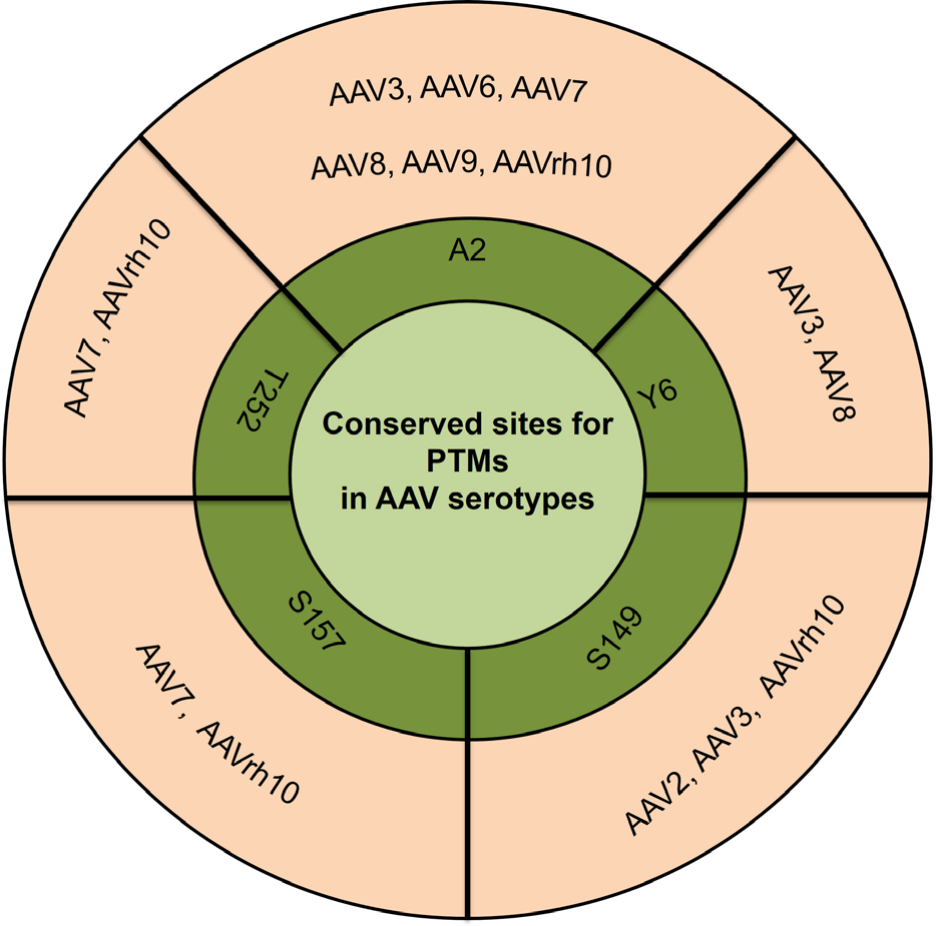
Conservation of post‐translational modifications identified in serotypes AAV2, AAV3, AAV6, AAV7, AAV8, AAV9 and AAVrh10.

Our analysis also demonstrated that a vast majority of PTMs in AAV capsid occurred at highly conserved residues. For e.g., the acetylation at A2 residue in AAV3 was a conserved modification seen in multiple serotypes such as AAV6, AAV7, AAV8, AAV9, and AAVrh10. Jin *et al*. 16 had identified a similar acetylation PTMs in AAV serotypes AAV7, AAV9, and AAVrh10 at residue A2. Similarly, the Y6 residue in AAV3 that was phosphorylated was also conserved in AAV8. A complete list of experimentally detected PTMs that were identified in more than one serotype is presented in Fig. 4. These experimental data suggest that PTMs at these largely evolutionarily conserved residues are observed in multiple serotypes and are likely to be important for the capsid structure and function.

### Validation of select PTMs on AAV capsid proteins

#### Ubiquitination

**Figure 5:**
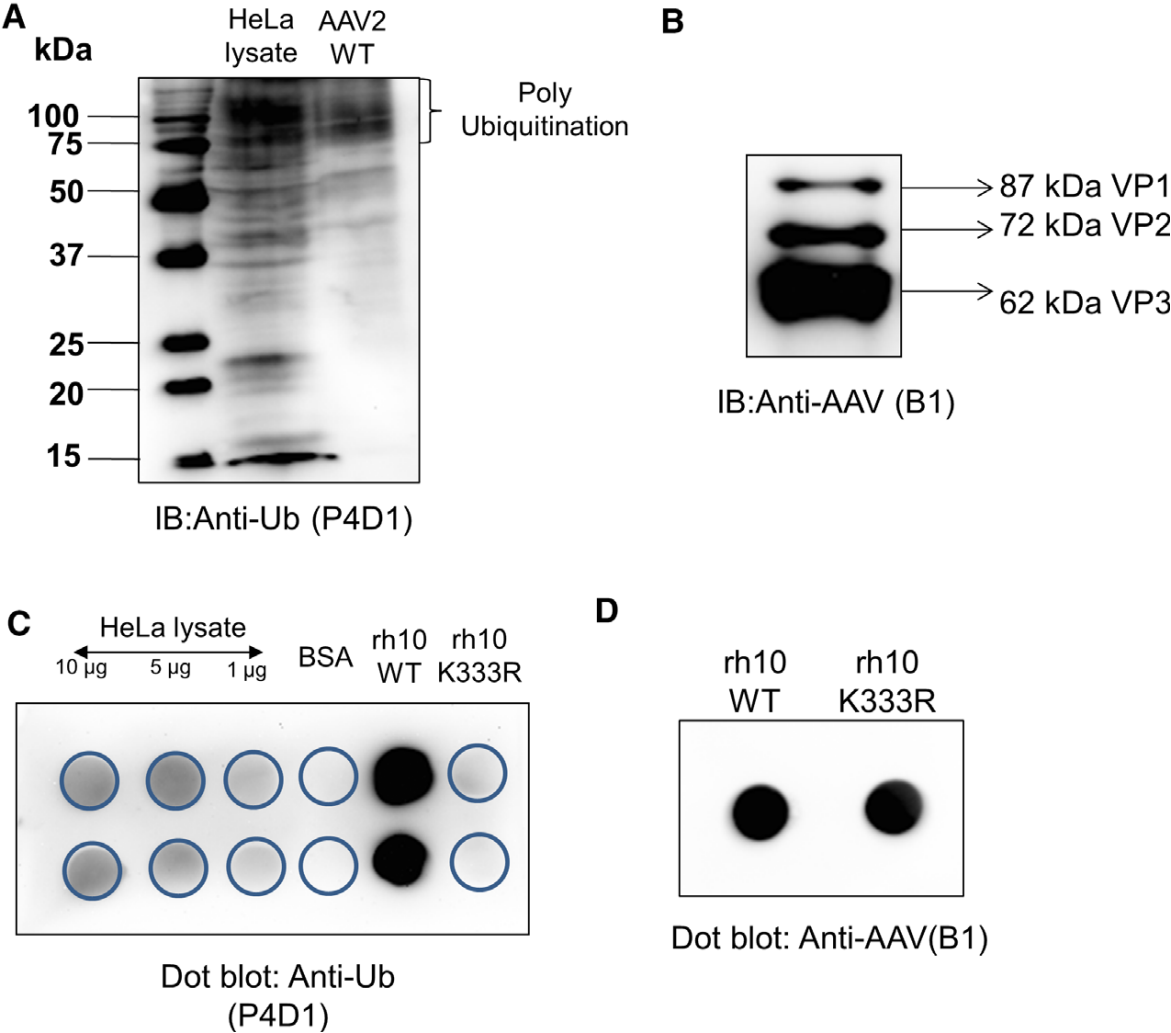
Western blot analysis to detect (A) total ubiquitination on AAV2 vectors and (B) VP1‐3 capsid proteins of AAV2 vectors. Dot‐blot analysis was performed to detect (C) native levels of ubiquitination on AAVrh10‐wild‐type (WT) or AAVrh10‐K333R mutant vectors and (D) VP1‐3 capsid proteins of AAVrh10 probed with an anti‐AAV B1 antibody as a loading control. Human cervical carcinoma cell (HeLa) lysate was used a positive control for these assays. The analysis was carried out independently twice and representative figures are shown.

To check, if AAV2‐wild‐type (WT) capsids are ubiquitinated, we performed a western blot analysis on AAV2 vectors packaged in AAV293 cells. A direct western blot analysis on packaged vectors showed the presence of ubiquitination, but in low abundance in VP1‐3 capsid proteins (Fig. 5A,B). The variation in the levels of ubiquitination observed here from earlier studies that have demonstrated higher levels of ubiquitination of AAV2 37, is probably due to the difference in techniques employed. While we have assessed for the native levels of ubiquitinated capsids in freshly packaged AAV2 vectors, previous studies have employed *in vitro* ubiquitination assays on AAV2 either directly 36 or after AAV isolation from infected cell lines by immunoprecipitation based methods and their subsequent incubation in the presence of excess amounts of a ubiquitin substrate and ligases 35. To further confirm this, we utilized a previously reported AAVrh10‐K333R mutant that showed comparable levels of ubiquitination to AAVrh10‐WT vectors in an *in vitro* ubiquitination analysis with excess amount of ubiquitination enzymes 50. These vectors when analyzed for native levels of ubiquitination, directly after packaging by a dot‐blot analysis had a distinctly reduced ubiquitination profile from WT‐vectors (Fig. 5C,D). Our data suggest that native levels of the ubiquitination in AAV2 is significantly low. However, this is likely to be masked when AAV capsids are pre‐incubated with excess of ubiquitination reagents.

#### Glycosylation

As described earlier, the detection of N‐glycosylation on AAV2 capsids by LC/MS, was further supported by our MALDI‐TOF based screening of total AAV2 capsids that identified multiple glycan moieties, as highlighted in Table 1. To understand the phenotypic effect of functional N‐glycan linked site on AAV2 capsids, one of the amino acid residue (N253) characterized to be N‐glycosylated by LC/MS was abolished to develop a mutant AAV2 vector (N253Q). Interestingly, the mutant AAV2 (N253Q) capsid, considerably reduced the vector yield in at least two different packaging experiments (average titer: 4.83 × 10^9^ vg·mL^−1^ vs. 5 × 10^11^ vg·mL^−1^) when compared to AAV2‐WT vectors. This suggests that the modification of N‐glycosylated residue (N253) in AAV2 capsid, results in loss of function of AAV2 capsid by compromising the vector assembly and stability, possibly through alterations to AAP.

#### SUMOylation

**Figure 6:**
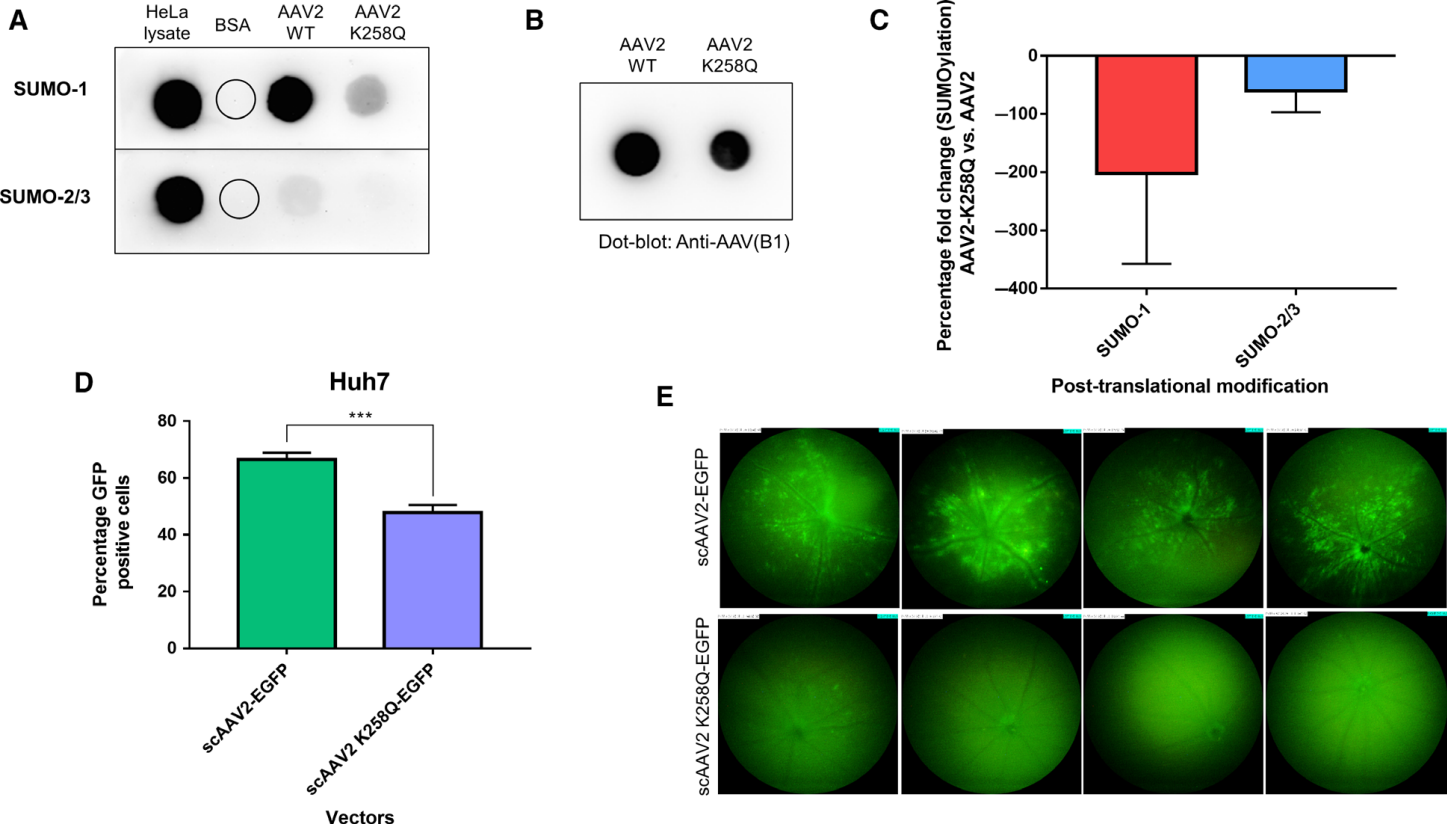
Characterization and validation of SUMOylation‐site modified AAV2 vectors. (A) Dot‐blot analysis for SUMO‐1 and SUMO‐2/3 proteins in AAV2‐wild‐type (WT) and AAV2‐K258Q vectors. HeLa cell lysate was used as positive control and bovine serum albumin (BSA) was used as a negative control for the assay (B) AAV capsid specific antibody (Anti‐AAV, B1) was used as a loading control for AAV2‐WT and AAV2‐K258Q vectors (C) Densitometric analysis of SUMO‐1 and SUMO‐2/3 dot‐blot on AAV2‐WT and AAV2‐K258Q, normalized to loading control (Anti‐AAV, B1 antibody) Two independent experiments were performed, and the data is a representation from a single analysis. (D) *In vitro* infectivity of AAV2‐WT and AAV2 K258Q mutant vectors in Huh7 cells. Flow cytometry analysis for GFP expression was performed 48 hr. post infection with the AAV2 vectors. Data are a mean of two independent experiments with three replicates included in each experiment. Error bars represent SD, ***P < 0.001. (E) Ocular gene transfer efficiency of AAV2‐WT and AAV2‐K258Q vectors was assessed by fundus imaging of murine eyes, 2 weeks after intravitreal gene transfer with vectors. The standard fundus imaging in the Micron IV system (Phoenix Research Laboratories, Pleasanton, CA, USA) was performed with a mouse objective and a field of view at 50° (1.8‐mm diameter). The images demonstrate GFP expression from representative eyes (*n* = 4) from a total of six eyes administered for each group. All the images were captured at a uniform exposure setting for both the groups [Intensity at maximum, gain at 18 db (decimal gain), frame rate at 2 fps (frames per second)].

To confirm the presence of a SUMOylation (2/3) PTM site on AAV2 capsid, the K258 residue was modified by site‐directed mutagenesis to generate a mutant vector (AAV2‐K258Q) containing EGFP as the transgene. The modification of the SUMO‐2/3 site (K258Q) did not majorly affect the vector yield (3 × 10^11^ vg·mL^−1^ vs. 5 × 10^11^ vg·mL^−1^) when compared to AAV2‐WT vectors. Furthermore, to characterize the extent of SUMOylation in AAV2‐K258Q mutant vectors, a dot‐blot assay was performed. Since Lysine (K) can be a potential target for either SUMO‐1 or SUMO‐2/3 proteins, a qualitative assessment for both SUMO‐1 and SUMO‐2/3 proteins were performed. As can be seen in Fig. 6A, a relatively less level of SUMO‐2/3 and SUMO‐1 protein was detected in AAV2‐K258Q mutant vector in comparison to AAV2‐WT vectors. Interestingly, the levels of SUMO‐1 proteins was more predominant than SUMO‐2/3 modification in both the test samples as reported previously 51. Vectors probed with Anti‐AAV antibody (B1) was used as loading controls for both the WT and mutant vectors tested (Fig. 6B). Furthermore, densitometric analysis affirms the reduced levels of SUMOylation in AAV2‐K258Q (Fig. 6C). As can be seen in this analysis, there is a considerable difference in dot size, which is likely due to saturated exposure for some conditions during image acquisition. These data show that abolishing a SUMO‐2/3 site by an amino acid substitution can reduce the total SUMO protein levels (both SUMO‐1 and 2/3) in AAV2 capsids.

Furthermore, to study the functional phenotype of AAV2‐K258Q mutants developed, an *in vitro* transduction assay was performed in Huh7 cells. As shown in Fig. 6D, AAV2‐K258Q vectors demonstrated a reduced transduction (~ 28%) after 48 hr. when compared to AAV2‐WT vectors, in concordance with a previous report 49. We then evaluated their *in vivo* transduction ability in the retina, by intravitreal administration of AAV2‐WT and AAV2‐K258Q vectors in C57BL/6 mice at lower doses (3 × 10^8^ vgs/eye). Fundus imaging, after 2 weeks of vector injections, showed negligible EGFP fluorescence in AAV2‐K258Q administered eyes when compared to AAV2‐WT vector administered control eyes (Fig. 6E). This suggests that the elimination of a functional SUMOylation site in AAV2 vector, may affect its transduction efficiency both *in vitro* and *in vivo*.

## Discussion

The initiation and progression of AAV infection in a host cell is largely contingent upon an evolutionarily fine‐tuned capsid interaction with the host cells. A further layer of complexity that remains largely unexplored as in the case of recombinant vectors such as AAV, is the profile of PTM's acquired by these vectors during its *in vitro* packaging. It is likely that these modifications are dynamic, depending on the cell type and the AAV serotype. It is conceivable that such PTMs modulate complex cellular pathways to promote viral transduction or on the contrary serve as danger signals that prime host defense. A thorough understanding of the global profile and distribution of PTMs in AAV capsids in general and in a serotype specific manner in particular, is likely to facilitate mapping of molecular features and regulatory nodes of AAV infection. Significantly, chromatographic and mass spectrometry based analysis of other viruses such as Adenovirus 27, human BK virus 52, Vaccinia virus 53, and Influenza A and B virus 54 have yielded unique structural and functional insights into their life cycle. For e.g. analysis of phophoproteome of influenza viruses have deduced the effect of specific PTMs in viral entry and exit 54, while a global PTM screening carried out in human BK Polyoma virus revealed the importance of VP1 capsid phosphorylation in receptor binding 52. In case of AAV, LC/MS based analysis of AAV2 had provided preliminary evidence of glycosylated capsid, but this was not conclusively established 42. Similarly, screening of select AAV serotypes 16 identified acetylation as a major PTM. However, the precise distribution and role of other PTMs such as ubiquitination, SUMOylation in the AAV capsid are not experimentally characterized. Thus, we carried out a systematic analysis of PTMs in ten AAV serotypes (AAV1‐AAVrh10) and demonstrate for the first time a diverse range of previously uncharacterized PTMs in multiple AAV serotypes.

Interestingly, ~ 36% of PTMs detected in our analysis involved glycosylation of the capsid residues. Our MALDI‐TOF analysis initially revealed the presence of N‐linked glycans in AAV2 capsid (Table 1) and based on these observations, we further determined the glycan‐specific mass changes and the identity of substrate residues in other AAV serotypes. Given the versatility and diversity of the glycosylation as a PTM, it is not surprising that many studies have suggested that glycosylation has a broad impact on viral life cycle. Enveloped viruses (Influenza and HIV virus) utilize the cellular glycosylation machinery for their entry, replication, and survival in the host 55, 56, 57. In case of non‐enveloped viruses, a similar understanding of cellular glycosylation in the viral life cycle is limited. Nonetheless, it has been reported that Adenovirus contain glycosylated proteins including a mono O‐linked GlcNAc addition to the fiber protein 58, 59. Similarly, glycosylation is known to be important for the correct folding of Rotavirus capsid protein (VP7) 60. Here, pretreatment of cells with glycosylation inhibitors such as tunicamycin and in combination with Brefeldin A led to a misfolding of the luminal protein VP7 and inter‐disulfide bond aggregation. The role of cellular glycosylation on the transduction process is known in case of murine leukemia retrovirus, in which the authors demonstrated that the inhibition of cellular glycosylation, enhanced the transduction of vectors by up to 200‐fold, particularly in cells which were initially resistant 61.

In case of AAV vectors, the role of cellular glycosylation is contentious. In 2006, Murray *et al*. 42 reported the absence of glycosylation in AAV2 capsid protein when packaged in HeLa cells at a detection limit of 10% of VP1 protein. AAV capsid screening by Jin *et al*. demonstrated absence of glycosylation of the capsids 16. A more recent study has identified glycosylation of N499 residue in AAV8 capsid 46. Due to a combination of limited sensitivity of various platforms used and the sizeable capsid proteins, a minimal glycosylation of viral capsid is likely to be missed, as suggested earlier 42. Several AAV serotypes utilize glycans as their cellular receptors and co‐receptors. AAV1 and AAV6 utilize sialic acid 62, AAV2 utilize heparan sulfate proteoglycan 11, and AAV9 use N‐linked galactose as primary receptors 10 for attachment on the cell surface. However, glycosylation of capsids is an important PTM known to protect cleavage of viral proteins and masks the recognition and neutralization of viral capsids 63 and thus their further characterization is likely to be rewarding. Also, in contrast with our MALDI‐TOF data in AAV2 capsid, LC/MS analysis demonstrated presence of total 11 N‐glycans and 8 O‐glycans on AAV2‐rh10 serotypes. It is possible that the reductive elimination of O‐glycans in AAV2 capsid is less effective to allow for its sensitive detection by MALDI‐TOF analysis. The detailed representation of the PTMs identified for all serotypes are represented in the linear VP1 protein sequence (Data Set S1).

**Figure 7:**
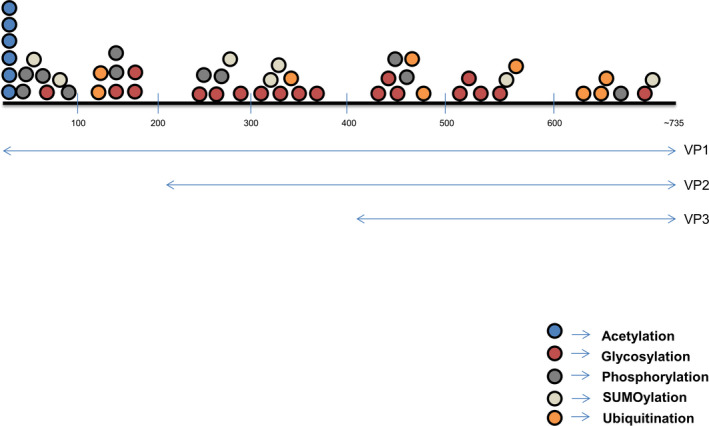
Distribution and type of post‐translational modifications on VP1, VP2, and VP3 capsid proteins of AAV1‐rh10 serotypes. Length of VP1, VP2, and VP3 capsid proteins are not to scale.

Our LC/MS data for AAV1‐rh10 demonstrated the presence of other PTMs like acetylation, phosphorylation, ubiquitination, and SUMOylation as shown in Fig. 7. Our data on acetylation of AAV capsids mirror previous findings in AAV5, 7, 9, and 10 serotypes 16. It is plausible that this acetylation is important for viral capsid degradation leading to possibly its un‐coating in the cell as postulated earlier 64. Alternatively, these acetylation signatures could act as a trigger for host cellular ubiquitination of the viral capsid during its cytoplasmic trafficking 64. The presence of ubiquitination and phosphorylation on AAV capsids was expected as several studies 35, 37, 49 have highlighted their role during viral entry or the intracellular trafficking process. Indeed, mutagenesis of several *in silico* predicted phosphorylation sites at serine, threonine, or tyrosine residues or ubiquitination targets (lysine residues) have improved the transduction efficiency of multiple AAV serotypes 36, 50. Our study also identified for the first time, SUMOylation targets in AAV2, AAV5, AAV7, AAV9, and AAVrh10 due to the mass difference of +600.25 (SUMO‐1) and +599.27 (SUMO‐2/3). SUMOylation is a reversible attachment of a small ubiquitin related modifier (SUMO) on the side chain amine group of a lysine aminoacid. Interestingly, several studies 65, 66, 67, have highlighted the independent as well as interdependent functions of SUMOylation with ubiquitination 68. A previous report 69 had demonstrated that small RNA inhibition of SUMO (E1/E2 enzymes of SUMOylation pathway) protein improves viral transduction. SUMOylated peptides are also immunogenic and serve as precursor signals for cellular immune response 70. Based on these observations, the study of unique SUMOylation targets defined in some of the AAV serotypes here is likely to furnish additional regulatory mechanisms that control the intracellular fate of AAV capsid.

To further validate the LC/MS derived peptide hits with PTMs, we searched the peptide sequences in BLAST and SWISSPROT database to ensure that these sequences are not derived from any cellular or sample preparative contaminants. While the 52 peptides described here were specific to AAV capsid sequence, an additional four peptides (data not shown) aligned to mammalian proteins, so these four hits were not included in our data analysis. In addition, a limited screen of AAV2 capsid by western blot analysis for ubiquitination and MALDI‐TOF analysis for glycosylation independently validated our findings.

To understand and validate the importance of PTM modifications such as SUMOylation and glycosylation in AAV capsid, mutant vectors modified at specific PTMs in AAV2 background were developed. AAV2 mutants (AAV2‐K258Q) modified at a SUMO‐2/3 protein binding site, showed considerable reduction in SUMO protein levels than wild‐type vectors. Further these SUMOylation site modified vectors had significantly lower transduction *in vitro* and *in vivo*. We speculate that the modest transduction seen with the AAV2‐K258Q vector may be due to a change in SUMOylation status of the capsid or due to perturbation of its interaction with certain critical amino acids involved in multiple steps of the viral life cycle such as induction of less efficient entry pathways 69. Similarly the N‐glycosylation mutant (AAV2‐N253Q) showed a reduced packaging efficiency in comparison to AAV2‐WT vectors. The mutation of N253 residue leads to a missense codon change in AAP (Threonine→Arginine), thus possibly affecting the vector yield. Alternatively, the glycosylation PTM at this site may be critical for capsid protein processing steps including their maturation, folding, or assembly. Further detailed studies are needed to pinpoint the exact mechanisms that contribute to a loss of function with the SUMOylation or glycosylation site modified AAV2 vectors.

Our study has limitations. PTMs on the same protein is variable as proteins mature differently in various cell lines 71. The PTM data obtained from AAV capsid proteins in our study was from a single packaging cell line, AAV293 cells. It is plausible that there will be considerable heterogeneity in the type and abundance of PTMs on AAV capsid between different methods of packaging or if the packaging cell line is varied 72, 73. Furthermore, our experimental setup did not facilitate the measure of PTMs at a monomer level on VP capsid protein or their temporal impact on capsid assembly process. In addition, it remains to be explored if this diversity of PTMs on the capsids leads to a phenotypic heterogeneity in AAV life cycle. Furthermore, we identified several other residues containing PTMs from our analysis, but are not reported here due to poor y‐ion and b‐ion fragmentation. Nonetheless, it indicates that multiple other PTMs could potentially coat AAV capsids, but in low abundance.

## Materials and methods

### Generation of AAV vectors

Self‐complementary AAV1 through AAVrh10 serotype wild‐type (WT) vectors or their select mutant variants containing the enhanced green fluorescence protein (EGFP) gene under the control of a chicken β‐actin promoter and bovine growth hormone poly (A) signal were generated as described previously 36. Briefly, 20 numbers of 150 mm dishes, 80% confluent with AAV‐293 cells were transfected with AAV (rep/cap), transgene (pds.AAV2‐EGFP), and AAV‐helper (p.helper) plasmids. Cells were collected 68–72 h later, lysed and treated with benzonase (25 units·mL^−1^; Sigma‐Aldrich, St Louis, MO, USA). Furthermore, vectors were purified by iodixanol gradient ultracentrifugation (OptiPrep; Sigma‐Aldrich) followed by column chromatography (HiTrap SP or Q column; GE Healthcare Life Sciences, Pittsburgh, PA, USA). The purified vectors were concentrated to a final volume of 0.5 mL in phosphate‐buffered saline (PBS), using Amicon Ultra 10K centrifugal filters (Millipore, Bedford, MA, USA). Physical titers of the vectors in vector genomes (vg)·mL^−1^ were assessed by a quantitative (q)PCR as described elsewhere 74.

### Enzymatic digestion of AAV capsids

About 25 μg of protein extract from AAV vectors was reduced with 5 mm DTT for 30 min at 60 °C and alkylated with 10 mm Iodoacetamide for 30mins in dark, at room temperature. Samples were digested overnight at 37 °C using trypsin in 1:25 enzyme substrate ratio. Digested samples were dried using a Speed Vac and stored at −20 °C till further processing.

### Mass spectrometry

Digested samples were further purified by the C18 cartridge (SepPak; Waters, Milford, MA, USA) and dried using Speed Vac. After the samples were resuspended in 5% Acetonitrile, 0.1% formic acid, LC/MS was performed on either Eksigent Nano UPLC 425 (AB Sciex, Framingham, MA, USA) in conjunction with Triple TOF 5600+ MS (AB Sciex) or EASY‐nLC 1000 system (Thermo Fisher Scientific, Waltham, MA, USA) coupled to QExactive MS (Thermo Fisher Scientific) equipped with nano‐electrospray ion source.

For data acquisition on Triple TOF, initial separation was achieved on LC column packed with the C18 material (2.7 μm particle), sized 0.5 × 100 mm. The total flow rate was 20 μL·min^−1^. The peptides were eluted using solvent A (water with 0.1% formic acid) and solvent B (acetonitrile with 0.1% formic acid) over 40mins. The raw data were acquired by using Analyst^®^ Software version 1.6. (AB Sciex).

For data acquisition on QExactive, 1 μg of the peptide mixture was loaded on a precolumn and resolved using 15 cm Pico‐Frit filled with 1.8 μm C18‐resin (Dr. Maisch GmbH, Ammerbuch, Germany). The peptides were loaded with Buffer A and further passed through Buffer‐B at a flow rate of 300 nl·min^−1^ in a 0–40% gradient of 95% acetonitrile/0.1% formic acid, for 105 min. The QExactive was run using the Top10 HCD data‐dependent acquisition mode with a full scan resolution of 70 000 at m/z 400. Furthermore, the MS/MS scans were obtained at a resolution of 17 500 at m/z 400. Lock mass option was enabled for polydimethylcyclosiloxane (PCM) ions (m/z = 445.120025) during the run, for performing the internal recalibration.

### MALDI‐TOF/TOF analysis

For N‐glycan detection, ~500 μg of protein extract from AAV2 vectors was reduced and alkylated and digested. Samples were then treated with glycosidase enzyme PNGase F for deglycosylation at 37 °C for 24 h and purified by C18 cartridge. For O‐glycan detection, samples were prepared by permethylation using methyl iodide. Phase separation was carried out with chloroform and the samples were recovered and purified by C18 cartridge. Samples for both N‐glycan and O‐glycan analysis were step eluted with 15%, 35%, 50%, and 75% Acetonitrile (ACN) using SEP PAK C 18 columns, then dried in Speed Vac and resuspended in 10 μL of methanol and DHB mixture analyzed using MALDI‐TOF/TOF 5800 (AB Sciex) in positive mode.

### Data analysis

PEAKS^®^ Studio 8.0 (Bioinformatics Solutions Inc., Waterloo, ON, Canada) software was used for the data analysis. Peptide identification was then performed by CID/HCD‐based MS/MS of the selected precursors which also reveals the site of modifications. For protein/peptide identification MS/MS data were searched against the amino acid sequences retrieved from NCBI database (Table 7). The search was set up for full tryptic peptides with a maximum of three missed cleavage sites. Acetylation, Phosphorylation, Ubiquitination, SUMOylation, and Glycosylation were included as variable modifications. The precursor mass tolerance threshold was at 10 ppm and in addition, 0.05 Da was set as the maximum fragment mass error. The significance threshold of the ion score was calculated based on a false discovery rate of ≤ 1%.

**Table 7:** Accession numbers of reference AAV database sequences retrieved from NCBI [https://www.ncbi.nlm.nih.gov/nuccore/?term=] for PEAKS Studio analysis.

Serotype	Accession id
AAV1	NC_002077.1
AAV2	NC_001401.2
AAV3	U48704.1
AAV4	NC_001829.1
AAV5	NC_006152.1
AAV6	AF028704.1
AAV7	NC_006260.1
AAV8	NC_006261.1
AAV9	AY530579.1
AAVrh10	AY243015.1

### Western blot analysis

Approximately, 1.42 × 10^10^ vg. of AAV2 vectors were resolved in a 10% denaturing gel and transferred into a PVDF membrane (Pall Corporation, New York city, NY, USA). Immunoblotting was performed with a mouse anti‐ubiquitin monoclonal antibody (P4D1) (Cell Signaling Technology, Danvers, MA, USA) or mouse anti‐AAV (B1) antibody used as a loading control (Fitzgerald, North Acton, MA, USA) and further probed with anti‐mouse IgG HRP secondary antibody (Abcam, Cambridge, UK). The immuno‐reactive bands were visualized using a chemiluminescence detection kit (Super signal West Pico PLUS kit, Thermo Scientific).

### Dot‐blot analysis

Dot‐blot screening was performed for ascertaining total levels of SUMOylation (SUMO‐1/2/3) on AAV2‐WT, AAV2K258Q capsids or the ubiquitination status of AAVrh10‐WT and AAVrh10‐K333R capsid proteins. For ubiquitination, ~ 5 × 10^8^ vg. of AAVrh10‐WT and AAVrh10‐K333R was spotted on activated PVDF membrane in equal volume. For SUMOylation, 5 × 10^9^ vgs. of AAV2‐WT and AAV2‐K258Q was spotted in the membrane in equal volume. For loading control, prior to spotting, vectors were preincubated with 0.4N NaOH at room temperature for 30 min. Furthermore, membranes were blocked in 5% BSA for 30 min. Membrane was then incubated with respective primary antibodies diluted in blocking solution for 1 h. After washing with Tris‐buffered Saline with 0.1% Tween 20 (TBST) thrice, membrane was incubated with HRP conjugated secondary antibody for 30 min. After adequate washing with TBST, blots were probed by using a chemiluminescence detection kit (Thermo Scientific). Dot‐blots were performed twice and representative images are shown. Antibodies used were, Anti‐SUMO‐1 (1 : 1000, Sigma‐Aldrich), Anti‐SUMO‐2/3 (1:1000, Cell Signaling Technology), Anti‐Ubiquitin (1:500, Cell Signaling Technology), Anti‐AAV(B1) (1:500, Fitzgerald), Anti‐mouse‐HRP conjugate (1:2500, Abcam), Anti‐rabbit‐HRP conjugate (1:500, Cell Signaling Technology). For densitometric analysis, intensities of immunoreactive spots from SUMO‐1 and SUMO2/3 blots of AAV2‐ K258Q was normalized to AAV2‐WT and their respective loading (B1) controls.

### Infectivity assay

Approximately, 5 × 10^4^ Huh7 cells were seeded in a 12‐well plate and incubated for 12 hr. in IMDM (Thermo Scientific) with 10% fetal bovine serum (Thermo Scientific) in humidified condition with 5% CO_2_. Cells were mock‐infected or infected with 5 × 10^3^ vg. of scAAV2‐EGFP or scAAV2 K258Q‐EGFP vectors. Forty‐eight hr. later, GFP expression was analyzed by flow cytometry (BD Accuri, BD Biosciences, Franklin Lakes, NJ, USA).

### Ocular gene transfer and imaging

Animal experiments were approved and performed according to the Institutional Guidelines for Animal Care at Indian Institute of Technology, Kanpur, India.

Eyes of C57BL6/J mice (*n = 6* eyes) were dilated by Phenylephrine + Tropicamide solution (Sunways India Pvt. Ltd. Mumbai, India). Mice were anesthetized by intraperitoneal injection of Ketamine (80 mg·kg^−1^) and Xylazine (12 mg·kg^−1^). For intravitreal administration, an opening was created at sclera near limbus by an insulin syringe, and 1 μL of AAV2‐WT or AAV2‐K258Q mutant vectors (3 × 10^8^ vg.) were injected through the same opening by Hamilton syringe fitted with 33‐gauge beveled needle, tobramycin (Sunways India Pvt. Ltd.) was applied to the eyes post injections. Fluorescence imaging was performed after 2 weeks of vector administration in Micron IV imaging system as per manufacturer's instructions (Phoenix Research Lab, Pleasanton, CA, USA). Briefly, eyes of C57BL6/J mice were dilated by Phenylephrine + Tropicamide solution (Sunways India Pvt. Ltd.). Mice were anesthetized by intraperitoneal injection of ketamine (80 mg·kg^−1^) and xylazine (12 mg·kg^−1^). Furthermore, 2.5% hypromellose (OCuSOFT, Rosenberg, TX, USA) was applied to the eye before imaging. Intensity was set at maximum and gain was set at 18 db., the frame rate was set at 2 fps for imaging of all the groups.

## Conflicts of interest

The authors declare no conflict of interest.

## Author contributions

BM, SM, SA performed the experiments. VK performed PTM analysis. BM, SM and GRJ wrote the manuscript. GRJ supervised the study.

## Supporting information


**Data Set S1.** Post‐translational modifications occurring on AAV2‐AAVrh10 serotypes are depicted in the linear VP1 sequence of respective serotypes [A‐I].
