# Supplementary material for: Post‐translational modifications in capsid proteins of recombinant adeno‐associated virus (AAV) 1‐rh10 serotypes
Source: FEBS J. 2019 Aug 1;286(24):4964–81. doi: 10.1111/febs.15013 (PMC7496479; doi:10.1111/febs.15013)
Supplement: Supplementary file 1 — Data Set S1. Post‐translational modifications occurring on AAV2‐AAVrh10 serotypes are depicted in the linear VP1 sequence of respective serotypes [A‐I]. [file FEBS-286-4964-s001.zip › febs15013-sup-0001-Data SetS1.pdf]

FEBS PRESS
science publishing by scientists
WILEY
The FEBS Journal
www.febsjournal.org

Post-translational modifications in capsid proteins of recombinant adeno-associated virus (AAV) 1-rh10 serotypes

Bertin Mary, Shubham Maurya, Sathyathithan Arumugam, Vikas Kumar and Giridhara R. Jayandharan

DOI: 10.1111/febs.15013

A

AAV2

1 MAADGYLPDWLEDTLSEGIRQWWKLKPGPPPPKPAERHKDDSRGLVLPGYKYLGPFNGLDKGEPVNEADA 70

71 AALEHDKAYDRQLDSGDNPYLKYNHADAEFQERLKEDTSFGGNLGRAVFQAKKRVLEPLGLVEEPVKTAP 140

141 GKKRPVEH**S**PVEPDSSSGTGKAGQQPARKRLNFGQTGDADSVPDPQPLGQPPAAPSGLGTNTMATGSGAP 210

211 MADNNEGADGVGNSSGNWHCDSTWMGDRVITTSTRTWALPTY**N**NHLY**K**QISSQSGASNDNHYFGYSTPWG 280

281 YFDFNRFHCHFSPRDWQRLINNNWGFRPKRLNFKLFNIQVKEVTQNDGTTTIANNLTSTVQVFTDSEYQL 350

351 PYVLGSAHQGCLPPFPADVFMVPQYGYLTLNNGSQAVGRSSFYCLEYFPSQMLRTGNNFTFSYTFEDVPF 420

421 HSSYAHSQSLDRLMNPLIDQYLYYLSRTNTPSGTTTQSRLQFSQAGASDIRDQSRNWLPGPCYRQQRVSK 490

491 TSADNNNSEYSWTGATKYHLNGRDSLV**N**PGPAMASHKDDEEKFFPQ**S**GVLIFG**K**QGSEKT**N**VDIEKVMIT 560

561 DEEEIRTTNPVATEQYGSVSTNLQRGNRQAATADVNTQGVLPGMVWQDRDVYLQGPIWAKIPHTDGHFHP 630

631 SPLMGGFGLKHPPPQILIKNTPVPANPSTTFSAAKFASFITQYSTGQVSVEIEWELQKENSKRWNPEIQY 700

701 TSNYNKSVNVDFTVDTNGVYSEPRPIGTRYLTRNL 735

→ **Acetylation**

→ **HexNAcylation**

→ **N-Glycan Core**

→ **Phosphorylation**

→ **SUMOylation**

→ **Ubiquitination**

B

AAV3

1 MAADGYLPDWLEDNLSEGIREWWALKPGVPQPKANQQHQDNRRGLVLPGYKYLGPGNGLDKGEPVNEADA 70

71 AALEHDKAYDQQLKAGDNPYLKYNHADAEFQERLQEDTSFGGNLGRAVFQAKKRILEPLGLVEEAAKTAP 140

141 GKKGAVDQSPQEPDSSSGVGKSGKQPARKRLNFGQTGDSESVPDPQPLGEPPAAPTSLGSNTMASGGGAP 210

211 MADNNEGADGVGNSSGNWHCDSQWLGDRVITTSTRTWALPTYNNHLYKQISSQSGASNDNHYFGYSTPWG 280

281 YFDFNRFHCHFSPRDWQRLINNNWGFRPKKLSFKLFNIQVRGVTQNDGTTTIANNLTSTVQVFTDSEYQL 350

351 PYVLGSAHQGCLPPFPADVFMVPQYGYLTLNNGSQAVGRSSFYCLEYFPSQMLRTGNNFQFSYTFEDVPF 420

421 HSSYAHSQSLDRLMNPLIDQYLYYLNRTQGTTSGTTNQSRLLFSQAGPQSMSLQARNWLPGPCYRQQRLS 490

491 KTANDNNNSNFPWTAASKYHLNGRDSLVNPGPAMASHKDDEEKFFPMHGNLIFGKEGTTASNAELDNVMI 560

561 TDEEEIRTTNPVATEQYGTVANNLQSSNTAPTTGTVNHQGALPGMVWQDRDVYLQGPIWAKIPHTDGHFH 630

631 PSPLMGGFGLKHPPPQIMIKNTPVPANPPTTFSPAKFASFITQYSTGQVSVEIEWELQKENSKRWNPEIQ 700

701 YTSNYNKSVNVDFTVDTNGVYSEPRPIGTRYLTRNL 736

→ **Acetylation**
→ **HexNAcylation**
→ **N-Glycan Core**
→ **Phosphorylation**
→ **SUMOylation**
→ **Ubiquitination**

C

AAV4

1 MTDGYLPDWLEDNLSEGVREWWALQPGAPKPKANQQHQDNARGLVLPGYKYLGPGNGLDKGEPVNAADAA 70

71 ALEHDKAYDQQLKAGDNPYLKYNHADAEFQQRLQGDTSFGGNLGRAVFQAKKRVLEPLGLVEQAGETAPG 140

141 KKRPLIESPQQPDSSTGIGKKGKQPAKKKLVFEDETGAGDGPPEGSTSGAMSDDSEMRAAAGGAAVEGGQ 210

211 GADGVGNASGDWHCDSTWSEGHVTTTSTRTWVLPTYNNHLYKRLGESLQSNTYNGFSTPWGYFDFNRFHC 280

281 HFSPRDWQRLINNNWGMRPKAMRVKIFNIQVKEVTTSNGETTVANNLTSTVQIFADSSYELPYVMDAGQE 350

351 GSLPPFPNDVFMVPQYGYCGLVTGNTSQQQTDRNAFYCLEYFPSQMLRTGNNFEITYSFEKVPFHSMYAH 420

421 SQSLDRLMNPLIDQYLWGLQSTTTGTTLNAGTATTNFTKLRPTNFSNFKKNWLPGPSIKQQGFSKTANQN 490

491 YKIPATGSDSLIKYETHSTLDGRWSALTPGPPMATAGPADSKFSNSQLIFAGPKQNGNTATVPGTLIFTS 560

561 EEELAATNATDTDMWGNLPGGDQSNSNLPTVDRLTALGAVPGMVWQNRDIYYQGPIWAKIPHTDGHFHPS 630

631 PLIGGFGLKHPPPQIFIKNTPVPANPATTFSSTPVNSFITQYSTGQVSVQIDWEIQKERSKRWNPEVQFT 700

701 SNYGQQNSLLWAPDAAGKYTEPRAIGTRYLTHHL 734

Acetylation

HexNAcylation

N-Glycan Core

Phosphorylation

SUMOylation

Ubiquitination

D

AAV5

1 MSFVDHPPDWLEEVGEGLREFLGLEAGPPKPKPNQQHQDQARGLVLPGYNYLGPGNGLDRGEPVNRADEV 70

71 AREHDISYNEQLEAGDNPYLKYNHADAEFQEKLADDTSFGGNLGKAVFQAKKRVLEPFGLVEEGAKTAPT 140

141 GKRIDDHFPKRKKARTEEDSKPSTSSDAEAGPSGSQQLQIPAQPASSLGADTMSAGGGGPLGDNNQGADG 210

211 VGNASGDWHCDSTWMGDRVVTKSTRTWVLPSYNNHQYREIKSGSVDGSNANAYFGYSTPWGYFDFNRFHS 280

281 HWSPRDWQRLINNYWGFRPRSLRVKIFNIQVKEVTVQDSTTTIANNLTSTVQVFTDDDYQLPYVVGNGTE 350

351 GCLPAFPPQVFTLPQYGYATLNRDNTENPTERSSFFCLEYFPSKMLRTGNNFEFTYNFEEVPFHSSFAPS 420

421 QNLFKLANPLVDQYLYRFVSTNNTGGVQFNKNLAGRYANTYKNWFPGPMGRTQGWNLGSGVNRASVSAFA 490

491 TTNRMELEGASYQVPPQPNGMTNNLQGSNTYALENTMIFNSQPANPGTTATYLEGNMLITSESETQPVNR 560

561 VAYNVGGQMATNNQSSTTAPATGTYNLQEIVPGSVWMERDVYLQGPIWAKIPETGAHFHPSPAMGGFGLK 630

631 HPPPMMLIKNTPVPGNITSFSDVPVSSFITQYSTGQVTVEMEWELKKENSKRWNPEIQYTNNYNDPQFVD 700

701 FAPDSTGEYRTTRPIGTRYLTRPL 724

- Acetylation
- HexNAcylation
- N-Glycan Core
- Phosphorylation
- SUMOylation
- Ubiquitination

E

AAV6

1 MAADGYLPDWLEDNLSEGIREWWDLKPGAPKPKANQQKQDDGRGLVLPGYKYLGPFNGLDKGEPVNAAD 70

71 AAALEHDKAYDQQLKAGDNPYLRYNHADAEFQERLQEDTSFGGNLGRAVFQAKKRVLEPFGLVEEGAKTAP 140

141 GKKRPVEQSPQEPDSSSGIGKTGQQPAKKRLNFGQTGDSESVPDPQPLGEPPATPAAVGPTTMASGGGAP 210

211 MADNNEGADGVGNASGNWHCDSTWLGDRVITTSTRTWALPTYNNHLYKQISSASTGASNDNHYFGYSTP 280

281 GYFDFNRFHCHFSPRDWQRLINNNWGFRPKRLNFKLFNIQVKEVTTNDGVTTIANNLTSTVQVFSDSEYQ 350

351 LPYVLGSAHQGCLPPFPADVFMIPQYGYLTLNNGSQAVGRSSFYCLEYFPSQMLRTGNNFTFSYTFEDVP 420

421 FHSSYAHSQSLDRLMNPLIDQYLYYLNRTQNQSGSAQNKDLLFSRGSPAGMSVQPKNWLPGPCYRQQRVS 490

491 KTKTDNNNSNFTWTGASKYNLNGRESIINPGTAMASHKDDKDKFFPMSGVMIFGKESAGASNTALDNVMI 560

561 TDEEEIKATNPVATERFGTVAVNLQSSSTDPATGDVHVMGALPGMVWQDRDVYLQGPIWAKIPHTDGHFH 630

631 PSPLMGGFGLKHPPPQILIKNTPVPANPPAEFSATKFASFITQYSTGQVSVEIEWELQKENSKRWNPEVQ 700

701 YTSNYAKSANVDFTVDNNGLYTEPRPIGTRYLTRPL 736

Acetylation

HexNAcylation

N-Glycan Core

Phosphorylation

SUMOylation

Ubiquitination

F

AAV7

1 MAADGYLPDWLEDNLSEGIREWWDLKPGAPKPKANQQKQDNGRGLVLPGYKYLGPFNGLDKGEPVNAADA 70

71 AALEHDKAYDQQLKAGDNPYLRYNHADAEFQERLQEDTSFGGNLGRAVFQAKKRVLEPLGLVEEGAKTAP 140

141 AKKRPVEPSPQRSPDSSTGIGKKGQQPARKRLNFGQTGDSESVPDPQPLGEPPAAPSSVGSGTVAAGGGA 210

211 PMADNNEGADGVGNASGNWHCDSTWLGDRVITTSTRTWALPTYNNHLYKQISSETAGSTNDNTYFGYSTP 280

281 WGYFDFNRFHCHFSPRDWQRLINNNWGFRPKKLRFKLFNIQVKEVTTNDGVTTIANNLTSTIQVFSDSEY 350

351 QLPYVLGSAHQGCLPPFPADVFMIPQYGYLTLNNGSQSVGRSSFYCLEYFPSQMLRTGNNFEFSYSFEDV 420

421 PFHSSYAHSQSLDRLMNPLIDQYLYYLARTQSNPGGTAGNRELQFYQGGPSTMAEQAKNWLPGPCFRQQR 490

491 VSKTLDQNNNSNFAWTGATKYHLNGRNSLVNPGVAMATHKDDEDRFFPSSGVLIFGKTGATNKTTLENVL 560

561 MTNEEEIRPTNPVATEEYGIVSSNLQAANTAAQTQVVNNQGALPGMVWQNRDVYLQGPIWAKIPHTDGNF 630

631 HPSPLMGGFGLKHPPPQILIKNTPVPANPPEVFTPAKFASFITQYSTGQVSVEIEWELQKENSKRWNPEI 700

701 QYTSNFEKQTGVDFAVDSQGVYSEPRPIGTRYLTRNL 737

Acetylation

HexNAcylation

N-Glycan Core

Phosphorylation

SUMOylation

Ubiquitination

G

AAV8

1 MAADGYLPDWLEDNLSEGIREWWALKPGAPKPKANQQKQDDGRGLVLPGYKYLGPFNGLDKGEPVNAADA 70

71 AALEHDKAYDQQLQAGDNPYLRYNHADAEFQERLQEDTSFGGNLGRAVFQAKKRVLEPLGLVEEGAKTAP 140

141 GKKRPVEPSPQRSPDSSTGIGKKGQQPARKRLNFGQTGDSESVPDPQPLGEPPAAPSGVGPNTMAAGGGA 210

211 PMADNNEGADGVGSSSGNWHCDSTWLGDRVITTSTRTWALPTYNNHLYKQISNGTSGGATNDNTYFGYST 280

281 PWGYFDFNRFHCHFSPRDWQRLINNNWGFRPKRLSFKLFNIQVKEVTQNEGTKTIANNLTSTIQVFTDSE 350

351 YQLPYVLGSAHQGCLPPFPADVFMIPQYGYLTLNNGSQAVGRSSFYCLEYFPSQMLRTGNNFQFTYTFED 420

421 VPFHSSYAHSQSLDRLMNPLIDQYLYYLSRTQTTGGTANTQTLGFSQGGPNTMANQAKNWLPGPCYRQQR 490

491 VSTTTGQNNNSNFAWTAGTKYHLNGRNSLANPGIAMATHKDDEERFFPSNGILIFGKQNAARDNADYSDV 560

561 MLTSEEEIKTTNPVATEEYGIVADNLQQQNTAPQIGTVNSQGALPGMVWQNRDVYLQGPIWAKIPHTDGN 630

631 FHPSPLMGGFGLKHPPPQILIKNTPVPADPPTTFNQSKLNSFITQYSTGQVSVEIEWELQKENSKRWNPE 700

701 IQYTSNYYKSTSVDFAVNTEGVYSEPRPIGTRYLTRNL 738

- Acetylation
- HexNAcylation
- N-Glycan Core
- Phosphorylation
- SUMOylation
- Ubiquitination

H

AAV9

1 MAADGYLPDWLEDNLSEGIREWWALKPGAPQPKANQQHQDNARGLVLPGYKYLGPGNGLDKGEPVNAADA 70

71 AALEHDKAYDQQLKAGDNPYLKYNHADAEFQERLKEDTSFGGNLGRAVFQAKKRLLEPLGLVEEAAKTAP 140

141 GKKRPVEQSPQEPDSSAGIGKSGAQPAKKRLNFGQTGDTESVPDPQPIGEPPAAPSGVGSLTMASGGGAP 210

211 VADNNEGADGVGSSSGNWHCDSQWLGDRVITTSTRTWALPTYNNHLYKQISNSTSGGSSNDNAYFGYSTP 280

281 WGYFDFNRFHCHFSPRDWQRLINNNWGFRPKRLNFKLFNIQVKEVTDNNGVKTIANNLTSTVQVFTDSDY 350

351 QLPYVLGSAHEGCLPPFPADVFMIPQYGYLTLNDGSQAVGRSSFYCLEYFPSQMLRTGNNFQFSYEFENV 420

421 PFHSSYAHSQSLDRLMNPLIDQYLYYLSKTINGSGQNQQTLKFSVAGPSNMAVQGRNYIPGPSYRQQRVS 490

491 TTVTQNNNSEFAWPGASSWALNGRNSLMNPGPAMASHKEGEDRFFPLSGSLIFGKQGTGRDNVDADKVMI 560

561 TNEEEIKTTNPVATESYGQVATNHQSAQAQAQTGWVQNQGILPGMVWQDRDVYLQGPIWAKIPHTDGNFH 630

631 PSPLMGGFGMKHPPPQILIKNTPVPADPPTAFNKDKLNSFITQYSTGQVSVEIEWELQKENSKRWNPEIQ 700

701 YTSNYYKSNNVEFAVNTEGVYSEPRPIGTRYLTRNL 736

→ **Acetylation**

→ **HexNAcylation**

→ **N-Glycan Core**

→ **Phosphorylation**

→ **SUMOylation**

→ **Ubiquitination**

I

AAV Rh10

1 MAADGYLPDWLEDNLSEGIREWWDLKPGAPKPKANQQKQDDGRGLVLPGYKYLGPFNGLDKGEPVNAADA 70

71 AALEHDKAYDQQLKAGDNPYLRYNHADAEFQERLQEDTSFGGNLGRAVFQAKKRVLEPLGLVEEGAKTAP 140

141 GKKRPVEPSPQRSPDSSTGIGKKGQQPAKKRLNFGQTGDSESVPDPQPIGEPPAGPSGLGSGTMAAGGGA 210

211 PMADNNEGADGVGSSSGNWHCDSTWLGDRVITTSTRTWALPTYNNHLYKQISNGTSGGSTNDNTYFGYST 280

281 PWGYFDFNRFHCHFSPRDWQRLINNNWGFRPKRLNFKLFNIQVKEVTQNEGTKTIANNLTSTIQVFTDSE 350

351 YQLPYVLGSAHQGCLPPFPADVFMIPQYGYLTLNNGSQAVGRSSFYCLEYFPSQMLRTGNNFEFSYQFED 420

421 VPFHSSYAHSQSLDRLMNPLIDQYLYYLSRTQSTGGTAGTQQLLFSQAGPNNMSAQAKNWLPGPCYRQQR 490

491 VSTTLSQNNNSNFAWTGATKYHLNGRDSLVNPGVAMATHKDDEERFFPSSGVLMFGKQGAGKDNVDYSSV 560

561 MLTSEEEIKTTNPVATEQYGVVADNLQQQNAAPIVGAVNSQGALPGMVWQNRDVYLQGPIWAKIPHTDGN 630

631 FHPSPLMGGFGLKHPPPQILIKNTPVPADPPTTFSQAKLASFITQYSTGQVSVEIEWELQKENSKRWNPE 700

701 IQYTSNYYKSTNVDFAVNTDGTYSEPRPIGTRYLTRNL 738

→ **Acetylation**

→ **HexNAcylation**

→ **N-Glycan Core**

→ **Phosphorylation**

→ **SUMOylation**

→ **Ubiquitination**
